# *In silico* and pharmacokinetic studies of glucomoringin from *Moringa oleifera* root for Alzheimer's disease like pathology

**DOI:** 10.2144/fsoa-2023-0255

**Published:** 2024-05-15

**Authors:** Chintalapati Manogna, Thirumal Margesan

**Affiliations:** 1 Department of Pharmacognosy, SRM College of Pharmacy, SRM Institute of Science & Technology, Kattankulathur, 603203, Chengalpattu, Tamil Nadu, India

**Keywords:** Alzheimer, biological activity, glucomoringin, herbal medicine, *in silico*, molecular docking, *Moringa oleifera*, pharmacokinetic, phytotherapy, toxicity

## Abstract

**Aim:** The aim of this research is to investigate the potential of glucomoringin, derived from *Moringa oleifera*, as a therapeutic agent for Alzheimer's disease through *in silico* analysis. **Materials & methods:** This study employs *in silico* or computational methodologies, including pkCSM, Swiss ADME, OSIRIS^®^ property explorer, PASS online web resource and MOLINSPIRATION^®^ software, to predict the pharmacokinetic characteristics and biological activity of glucomoringin. **Results & conclusion:** Molecular docking indicates strong binding to *I-1β* and the pharmacokinetic profile shows cytochrome P450 enzyme inhibition, prompting further research for dosing strategies. Toxicological predictions affirm safety, while bioactivity assessments demonstrate versatility in modulating essential pathways. glucomoringin's potential for Alzheimer's treatment, emphasizing the need for additional empirical research.

## Plain language summary

Alzheimer's disease is a severe brain condition that affects millions of people worldwide. It impairs memory and thinking and there is no cure yet. Researchers are looking into alternative treatments, like using the moringa plant. Some compounds in moringa, such as glucomoringin, seem promising for Alzheimer's treatment. However, it's essential to be cautious and consult with a doctor before trying these treatments. The study used computer simulations to see how well glucomoringin could work for Alzheimer's. It found that glucomoringin might be good at reducing inflammation in the brain. It also looked at how the body processes glucomoringin and found that it seems safe and could be used as a treatment. However, these are just computer predictions and more research is needed to confirm if glucomoringin is effective for Alzheimer's. Alzheimer's is a complex disease and there's no one-size-fits-all treatment. So, it's important to work with doctors to find the best approach for each patient. In the future, Alzheimer's research will focus on early detection, personalized treatments and using technology to help patients. Public awareness and support for Alzheimer's patients and their caregivers are crucial and global collaboration is vital for progress in finding a cure.

## Abstract (plain language summary)

The study suggests that glucomoringin has strong binding affinity to *IL-1β*, an inflammatory molecule associated with Alzheimer's disease. The pharmacokinetic analysis reveals important details about glucomoringin's properties, including its inhibition of specific cytochrome P450 enzymes and its clearance rate. These insights are crucial for understanding how the compound may interact with other drugs and how it is metabolized in the body.

The study underscores the significance of global collaboration in Alzheimer's research and the importance of personalized care for individuals with the disease. It emphasizes the complexity of Alzheimer's and the need for multifaceted approaches to treatment.

Glucomoringin demonstrates a favorable toxicological profile, with no indications of mutagenicity, carcinogenicity, irritant effects, or reproductive effects. This highlights its potential as a safe therapeutic option.

While the *in silico* findings are promising, the study emphasizes the importance of conducting empirical research to validate the efficacy and safety of glucomoringin for clinical use.

Glucomoringin exhibits a good bioactivity score in several key drug targets, such as GPCR ligands, ion channel modulators, kinase inhibitors and nuclear receptors. This versatility suggests its potential for modulating various biological pathways.

The study is part of the broader exploration of herbal treatments for Alzheimer's disease. It highlights the need for caution and the importance of consulting with healthcare professionals when considering alternative therapies.

Overall, the study offers promising insights into glucomoringin's potential as a therapeutic agent for Alzheimer's disease, but further empirical research is necessary to confirm its effectiveness and safety.

Alzheimer's disease, a devastating neurological condition, profoundly impacts millions worldwide. As the leading cause of dementia in older adults, it gradually impairs cognitive functions and memory. Coined by Dr. Alois Alzheimer in 1906, the disease is marked by abnormal protein deposits, mainly amyloid plaques and tau tangles, in the brain [1]. This results in the gradual deterioration of brain cells, leading to a continuous decline in cognitive function and eventual loss of independence. Alzheimer's disease poses a considerable public health challenge, inflicting emotional distress on affected individuals and imposing burdens on healthcare systems and society at large. This introduction seeks to offer an overview of this complex and debilitating disease, exploring its causes, symptoms, diagnosis, treatment and ongoing research endeavors to enhance understanding and address the challenges it presents [2]. The escalating prevalence of Alzheimer's disease is a global concern, exacerbated by an aging population. As of our last update in September 2021, about 50 million individuals worldwide were living with Alzheimer's or a related dementia. This figure is anticipated to surge, potentially reaching 152 million by 2050 without effective interventions [3]. In 2021, the Alzheimer's Association estimated over 6 million Americans lived with Alzheimer's disease, with projections indicating a significant increase in the coming decades. Primarily age-related, the risk rises with age, affecting individuals in their 40s or 50s (early-onset) but is more common in those over 65 years of age (late-onset). Notably, Alzheimer's has a higher prevalence among women [4]. The gender difference in Alzheimer's prevalence is partly attributed to women's longer life expectancy. Regional variations in prevalence may result from genetic, environmental and lifestyle factors. Besides the personal and familial impact, Alzheimer's poses economic and societal challenges, straining healthcare systems and communities. Prevalence rates might have changed post-2021, with ongoing research focusing on early detection, prevention and more effective treatments. As of September 2021, Alzheimer's lacks a cure; primary treatments involve medications and non-pharmacological interventions to manage symptoms. Complementary therapies like herbal treatments are explored cautiously under healthcare guidance. Notable herbs studied for Alzheimer's include *Ginkgo biloba* [^5-7^], curcumin [^8-11^], ginseng [^12-15^], huperzine [^16-19^], coconut oil [^20-22^], etc. Caution is essential when considering herbal treatments, particularly in Alzheimer's disease. Consulting with a healthcare professional is crucial due to potential interactions with medications, varying effects between individuals and possible side effects [23].

*Moringa oleifera*, also known as the ‘drumstick tree’, is a plant native to Africa and Asia. Renowned for its nutritional and potential medicinal properties, it is rich in vitamins, minerals and bioactive compounds [24,25]. It is a plant that has gained attention due to its potential health benefits and it contains various bioactive compounds, including alkaloids [25]. Alkaloids, natural organic compounds, exhibit diverse pharmacological effects in the human body. Interest in exploring the potential benefits of moringa and its alkaloids for Alzheimer's disease exists, but research in this area is limited. Specific alkaloids, like moringine, have been studied for their potential to inhibit cholinesterase enzymes [26,27]. Cholinesterase inhibitors, employed in Alzheimer's treatment to boost acetylcholine levels crucial for memory and cognition, exhibit mild inhibition in some studies. However, uncertainty surrounds their efficacy in treating Alzheimer's. Moringa alkaloids may hold neuroprotective potential [28,29]. Studies propose that certain compounds in moringa may shield brain cells, mitigate inflammation and promote brain health, potentially aligning with the mechanisms implicated in Alzheimer's disease. Numerous alkaloids in moringa exhibit antioxidant properties [^30-32^] and anti-inflammatory properties [^33-35^]. While the antioxidant and anti-inflammatory properties of moringa alkaloids are promising for Alzheimer's disease due to their potential role in mitigating oxidative stress and inflammation, it's crucial to note that preliminary research is at an early stage. Further comprehensive studies, including clinical trials, are necessary to ascertain the safety and efficacy of moringa alkaloids specifically for Alzheimer's disease. Given the complexity of Alzheimer's and the multifaceted nature of its progression, treatment typically involves a combination of medication, lifestyle adjustments and supportive care. If considering the potential benefits of moringa or its alkaloids for Alzheimer's, consulting with a healthcare professional is essential for guidance and ensuring safe integration with evidence-based treatments. While promising research suggests a potential impact of moringa on Alzheimer's disease, these studies are preliminary and may not directly translate to effective human treatments. Rigorous clinical trials are needed to understand benefits, dosages and safety considerations. Consult a healthcare professional for exploring alternative or complementary treatments. Alzheimer's complexity requires collaboration with medical experts to develop the best care plan. Ongoing research is crucial and while herbal treatments hold promise, they should complement evidence-based medical interventions, not replace them. Work closely with a healthcare provider for a comprehensive care plan if dealing with Alzheimer's.

*In silico studies*, a key aspect of modern scientific inquiry, revolutionize the exploration and understanding of complex systems. Rooted in computational methods, this innovative approach enables the simulation, modeling and prediction of diverse phenomena in fields such as biology, chemistry, materials science and engineering. Derived from the Latin phrase meaning ‘in silicon’, it symbolizes the shift from traditional experiments to digital simulations. These computational investigations utilize the vast processing power of computers to uncover patterns, streamline research and enhance our understanding of principles. They play a crucial role in designing drugs, materials and technologies more efficiently. This overview explores the applications and advancements in *in silico* studies, emphasizing its pivotal role in shaping 21st century scientific research [36,37]. Computational methods in *in silico* studies are becoming pivotal in Alzheimer's research, aiding in comprehending the disease, drug discovery and treatment development. These studies efficiently handle extensive data, predict outcomes and guide experimental research. Yet, while these approaches offer valuable insights and expedite research, they must be coupled with *in vitro* and *in vivo* studies to authenticate findings and create effective Alzheimer's treatments.

## Materials & methods

### Molecular docking analysis

The crystal structures, including 1F4G, 3IG5, 3E0U, 15EA, 6FN8, 5IKR, 1ITB, 1ALU, 1NFK and 2AZ5 were extracted from the Protein Data Bank (PDB) (http://www.rcsb.org). Subsequently, the PDB structures were refined by removing the inhibitor ligands and any other associated chains. The proteins utilized in this research were designed with the assistance of MMV and AutoDock. Following this initial design phase, a second round of enhancements was carried out on the modified proteins. This involved stabilizing the ionized and tautomeric states of the amino acid residues, necessitating the removal of water molecules and the addition of hydrogen atoms. To maintain the updated protein structure for further docking studies, a PDB file was generated. The optimization of the proteins in this study was accomplished using the Molegro molecular viewer. Adjustments to the bond order were made after the removal of water and covalently attached ligands. Charge and protonation states were assigned and the energy of the system was minimized using the molecular mechanics force field. For the molecular docking simulations, the AutoDock program was employed. This program automatically computed Gasteiger charges and assessed the ligand's rotatable bonds to generate multiple conformers for the docking process. Receptor grids were constructed based on designated receptor points and grid boxes were created using the receptor grids' axes as the domain. The macromolecule was positioned centrally within the grid. Several types of maps were generated using Autogrid 4. All molecular docking simulations were conducted using the Lamarckian genetic algorithm. The docking procedure was set to include 50 iterations, 150 subjects, 2.5 million assessments and 27,000 generations. Biovia Discovery Studio 2021 was utilized to import the docking snapshots and the resulting docked structures were exported to pdbqt format. Subsequently, Biovia Discovery Studio 2021 was employed to visualize the docking results, which revealed the presence of hydrogen and hydrophobic interactions at the inhibitor sites of the docked targets [^38-44^].

### Pharmacokinetic assessment

Extracting data from the PubChem server unveiled the standard chemical structure of glucomoringin. Comprehensive pharmacokinetic investigations were carried out using pkCSM and Swiss ADME. The ADMET profile was obtained from the host computer based on canonical smiles. Both pkCSM and Swiss ADME furnish insights into the pharmacokinetics (PK), pharmacodynamics (PD) and toxicology (toxicity) of the drug. PkCSM, as a web-based application, facilitates the examination of the pharmacokinetic characteristics of pharmaceutical compounds. The pkCSM software was employed to assess glucomoringin physicochemical attributes [45,46]. By evaluating their binding affinities and pharmacokinetic characteristics, we pinpointed the top-docked compounds, signifying their potential as therapeutic candidates. However, when employing effective strategies in drug design, development and discovery, it is crucial to take into account a range of critical pharmacokinetic factors and ADMET attributes, including absorption, distribution, metabolism, excretion and toxicity. To gain insights into the physicochemical properties and ADMET attributes of the substance under examination, an extensive investigation was conducted using online web-based tools. glucomoringin underwent an analysis based on Lipinski's rule of five, which identifies five essential physicochemical features that significantly influence a molecule's effectiveness, safety and metabolism. It was observed that there were violations of LogP across all three Consensus LogPo/w implementations tested (WLOGP, XLOGP3 and LOGP). The logarithm of the partition coefficient between n-octanol and water, representing lipophilicity values between -0.7 and +5.0, was determined using atomistic and topological implementations of Moriguchi's topological approach, including XLOGP3, WLOGP and MLOGP

### Toxicological modeling & simulation

In order to mitigate potential complications during the process of discontinuing a drug, which could include issues like organ system failure or damage, it is imperative to conduct toxicological assessments. The OSIRIS property explorer software was employed, utilizing PubChem structures, to assess the compounds' toxicity profiles. These substances are categorized on a color-coded scale according to their likelihood to induce cancer, mutations, irritation, reproductive effects and their potential as pharmaceutical agents. An overall drug score, a drug-likeness score and TPSA (topological polar surface area) were computed based on the following toxicity criteria: substances with high risk are denoted in red, those with medium risk are in yellow and those with low risk are depicted in green [44].

### Analysis of glucomoringin biological activity

The PASS (prediction of action spectra for substances) tool was employed to predict the biological actions of the medications. It anticipates the biological activity spectrum of a drug based solely on its structural formula, relying on the correlation between structure and behavior. The capacity of a chemical compound to exhibit pharmacological or biological effects is of paramount importance, as it unveils potential applications for the compound in the field of medicine. Furthermore, the potential adverse effects of these drugs were also foreseen with the assistance of the PASS ONLINE tool. Biologically active compounds were assessed using the web-based PASS program, which has the capability to forecast over 300 distinct pharmacological activities and biological pathways. These predictions are rooted in the structural formula of the substance and they hold the potential to uncover new pharmaceutical targets based on the gathered information [47,48].

### Molecular property analysis

The chosen drugs underwent *in silico* testing with the MOLINSPIRATION software to assess drug similarity and forecast their bioactivity. A higher score indicates an increased likelihood that the molecule in question will exhibit activity. A chemical with a bioactivity score greater than 0.00 is deemed to possess noteworthy biological properties. Scores falling within the range of -0.50 to 0.00 on the bioactivity scale are considered highly active, while values below -0.50 are categorized as inactive [49].

## Results

### Molecular docking assessment of glucomoringin

**Figure 1.**
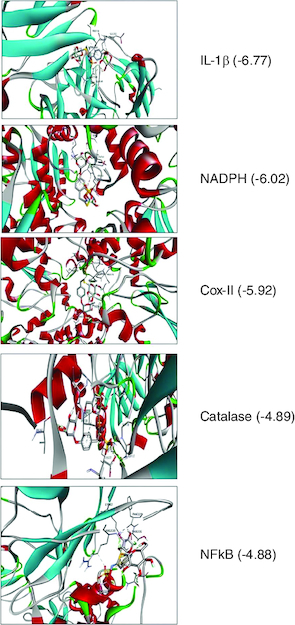
Docking assessment of glucomoringin with multiple targets of Alzheimer.

To determine whether glucomoringin might directly bind to the chosen genes, a ligand binding simulator was run before the experimental study. When comparing Alzheimer's biomarkers, oxidative stress associated protein catalase, glutamate cysteine ligase, glutathione peroxidase, NADPH quinone dehydrogenase, superoxide dismutase. Other targets inflammatory receptors include *COX-II*, *I-1β*, *IL-6*, *NF-κB*, *TNF-α* stand out as crucial. glucomoringin showed the highest binding energy in *IL1β* (PDB: 1ITB = -6.77 kcal/mol) which was illustrated in Figure 1. Since molecular docking persists as a computer simulation approach used for determining the shape of a receptor ligand complex, the hypothesis desires to be tested empirically in future studies, but the data presented here suggests that glucomoringin might have a significant ability to directly connect with inflammatory molecules. The glucomoringin formed a hydrogen bond with amino acid residues LYS114, GLU202, MET148, ASN204, ASN108 and GLN149 for 1ITB. Each target ligand generated at least one hydrogen bond with active chemical residues, providing confidence to the accuracy and precision of the prediction made by the investigation. The outcomes of molecular docking simulations for selective receptor binding are displayed in Table 1. In most selected targets, a considerably increased number of amino acid residues engaged in hydrogen bonding and Van der Waals interactions was related with shorter hydrogen bond lengths (less than 3.0).

**Table 1.** Docking assessment of glucomoringin on Alzheimer.

Protein	PDB ID	Binding energy kcal/mol	Conventional hydrogen bonding interaction
*COX-II*	5IKR	-5.92	GLN149, GLU202, LYS114, ASN204, MET148, ASN108
*IL1beta*	1ITB	-6.77	GLN149, GLU202, LYS114, ASN204, MET148, ASN108
*IL-6*	1ALU	-3.29	GLN149, GLU202, LYS114, ASN204, MET148, ASN108
*NF-κB*	1NFK	-4.88	GLN149, GLU202, LYS114, ASN204, MET148, ASN108
*TNF alpha*	2AZ5	-4.68	GLN149, GLU202, LYS114, ASN204, MET148, ASN108
*Catalase*	1F4J	-4.89	GLN149, GLU202, LYS114, ASN204, MET148, ASN108
*Glutamate*	3IJ5	-4.46	GLN149, GLU202, LYS114, ASN204, MET148, ASN108
*Glutathione*	3E0U	-3.05	GLN149, GLU202, LYS114, ASN204, MET148, ASN108
*NADPH*	5EA2	-6.02	GLN149, GLU202, LYS114, ASN204, MET148, ASN108
*SOD*	6FN8	-2.23	GLN149, GLU202, LYS114, ASN204, MET148, ASN108

### Glucomoringin pharmacokinetic profile

Glucomoringin is an inhibitor of CYP1A2, CYP2C9, CYP2C19 and CYP3A4 enzyme and its clearance was calculated to be -0.018 ml/min/kg. The pkCSM database shows that the maximum safe daily intake of glucomoringin in humans is 0.358 mg/kg, LD_50_ was predicted to be 3750 mg/kg and the drug profile was listed in Table 2. The predicted toxicity class 5, average similarity and prediction accuracy was found to be 42.1% and 54.26%. Hepatotoxicity, carcinogenicity, mutagenicity, cytotoxicity predicted inactive but surprisingly immunotoxicity found to be active.

**Table 2.** Pharmacokinetic parameters of glucomoringin.

Property	Model name	Predicted value	Unit
Absorption	Water solubility	-2.398	Absorption
Absorption	Caco2 permeability	-0.726	Absorption
Absorption	Intestinal absorption (human)	4.094	Absorption
Absorption	Skin Permeability	-2.735	Absorption
Absorption	P-glycoprotein substrate	No	Absorption
Absorption	P-glycoprotein I inhibitor	No	Absorption
Absorption	P-glycoprotein II inhibitor	No	Absorption
Distribution	VDss (human)	-0.596	Distribution
Distribution	Fraction unbound (human)	0.671	Distribution
Distribution	BBB permeability	-1.391	Distribution
Distribution	CNS permeability	-4.061	Distribution
Metabolism	CYP2D6 substrate	No	Metabolism
Metabolism	CYP3A4 substrate	No	Metabolism
Metabolism	CYP1A2 inhibitior	No	Metabolism
Metabolism	CYP2C19 inhibitior	No	Metabolism
Metabolism	CYP2C9 inhibitior	No	Metabolism
Metabolism	CYP2D6 inhibitior	No	Metabolism
Metabolism	CYP3A4 inhibitior	No	Metabolism
Excretion	Total Clearance	0.358	Excretion
Excretion	Renal OCT2 substrate	No	Excretion
Toxicity	AMES toxicity	No	Toxicity
Toxicity	Max. tolerated dose (human)	1.194	Toxicity
Toxicity	hERG I inhibitor	No	Toxicity
Toxicity	hERG II inhibitor	No	Toxicity
Toxicity	Oral Rat Acute Toxicity (LD50)	2.382	Toxicity
Toxicity	Oral Rat Chronic Toxicity (LOAEL)	3.764	Toxicity
Toxicity	Hepatotoxicity	Yes	Toxicity
Toxicity	Skin Sensitisation	No	Toxicity
Toxicity	T.Pyriformis toxicity	0.285	Toxicity
Toxicity	Minnow toxicity	4.648	Toxicity

The chemical does not cause skin sensitivity, liver, or renal damage. It yields a favorable bioactivity score for therapeutic targets; this pharmacokinetic technique is trustworthy for use in drug development and pharmacokinetic analysis. Based on its pharmacokinetic profile, SwissADME concludes that glucomoringin has a low GI absorption and it's a p-glycoprotein I and II inhibitor. Table 3 lists the predicted ADMET properties. However, % intestinal absorption rate of 4.094% is significantly low. The permeability of glucomoringin via the blood-brain barrier was found to be -1.394 (log BB). However, its central nervous system (CNS) permeability was found to be -4.061 (log PS), indicating enhanced CNS penetration and the potential to exert its pharmacological effects. ADMET predictions showed that the calculated absorption properties (percent human intestinal absorption, skin permeability and BBB permeability) were not P-glycoprotein I and II inhibitors because they were below the threshold values. This demonstrates that glucomoringin has significant pharmacological properties. Glucomoringin physiochemical profile was appealing because its anticipated values were within the acceptable range.

**Table 3.** glucomoringin profile.

Physiochemical parameters	
Molecule	glucomoringin
Canonical SMILES	OCC1OC(SC(=NOS(=O)(=O)[O-])Cc2ccc(cc2)OC2OC(C)C(C(C2O)O)O)C(C(C1O)O)O.[K+]
Formula	C20H28KNO14S2
MW	609.66
Heavy atoms	38
Aromatic heavy atoms	6
Fraction Csp3	0.65
Rotatable bonds	9
H-bond acceptors	15
H-bond donors	7
MR	122.7
TPSA	281.77
**Lipophilicity**	
iLOGP	-7.71
XLOGP3	-1.88
WLOGP	-2.16
MLOGP	-3.27
Silicos-IT Log P	-3.65
Consensus Log P	-3.73
**Water solubility**	
ESOL Log S	-1.96
ESOL Solubility (mg/ml)	6.71E+00
ESOL Solubility (mol/l)	1.10E-02
ESOL Class	Very soluble
Ali Log S	-3.52
Ali Solubility (mg/ml)	1.85E-01
Ali Solubility (mol/l)	3.04E-04
Ali Class	Soluble
Silicos-IT LogSw	1.71
Silicos-IT Solubility (mg/ml)	3.11E+04
Silicos-IT Solubility (mol/l)	5.11E+01
Silicos-IT class	Soluble

### Toxicological prediction of glucomoringin

The toxicity of the investigated inhibitors was calculated using *in silico* methods. The physicochemical properties of the substance were determined with the help of OSIRIS Property Explorer. Exploring the glucomoringin toxicity profile before putting it through clinical trials is crucial. glucomoringin was shown to have no mutagenic, carcinogenic, irritant, or reproductive effects, according to the toxicity characteristics of the compounds tested. Most of the suggested inhibitors were found to have acceptable toxicity profiles, making them a viable therapy alternative. The OSIRIS property explorer granted the property a clean health bill concerning the aspects mentioned earlier, increasing the property's security. The TPSA of a molecule is the ultimate determinant of how well a drug is absorbed. It also helps the medicine get where it needs to go. OSIRIS property explorer calculated the compound's Topological Polar Surface Area (TPSA) to be 281.77. The drug-likeness recorded for this molecule was -7.74. If the results are positive, it means that the molecule has parts that are commonly seen in commercially available drugs. You get its essence when you combine a drug's molecular weight, toxicity risks and cLogP. The medications' fundamentals are essential for figuring out if a given chemical can meet the needs of a treatment. The drug score of glucomoringin was found to be 0.3 and found that non-toxic compound (Green) in toxicity analysis using OSIRIS software. TPSA, drug-likeness, overall drug score was generated.

### Bioactivity score assessment of glucomoringin

The Molinspiration online tool provides free online services to the internet chemistry community, such as the prediction of bioactivity score for the most important drug targets such as GPCR ligands (0.19), ion channel modulators (-0.17), kinase inhibitors (-0.19) and nuclear receptors (-0.10) and the calculation of critical molecular properties (polar surface area, logP, number of hydrogen bond donors and acceptors and others).

## Discussion

Alzheimer's disease is a neurodegenerative ailment that is incredibly widespread and has a catastrophic impact on the lives of millions of people all over the world. Despite the fact that there is now no known cure, the treatment focuses mostly on symptom control. The investigation of complementary and alternative treatments, such as those involving herbs, to improve cognitive health has garnered a growing amount of interest. The use of precision medicine is yet another promising approach being taken in Alzheimer's research. Alzheimer's disease is not a disease that affects everyone in the same way; rather, it has a variety of underlying causes and elements that contribute to its progression. Researchers are increasingly concentrating their efforts on developing medicines that are uniquely suited to the genetic and molecular profile of the patient. This individualized strategy tries to target the fundamental causes of the disease, such as amyloid plaques and tau tangles, in order to slow down or stop the progression of the disease. Early study indicates that the plant Moringa oleifera, which is high in alkaloids, may be useful in the treatment of Alzheimer's disease. A number of these alkaloids have features that include inhibition of cholinesterase, neuroprotection, antioxidant activity and anti-inflammatory action. Herbal remedies, such as moringa, have shown some promise in the treatment of Alzheimer's disease; nonetheless, they should not be used in place of evidence-based medicinal procedures. glucomoringin has a high concentration of An analysis of molecular docking was performed on Moringa oleifera since it is of interest as a chemical. An exhaustive investigation into the potential of glucomoringin within the setting of Alzheimer's disease using *in silico* experiments. Strong binding to interleukin-1 (-5.77 kcal/mol) was observed, which is suggestive of the possibility of direct interaction with inflammatory molecules. Inflammation is an essential component of Alzheimer's disease. The formation of hydrogen bonds with particular amino acid residues in the target provides evidence that the interaction is particularly robust. The pharmacokinetic profile of the chemical showed that it inhibited a number of cytochrome P450 enzymes and it reported having a decent clearance rate. The predicted safety statistics revealed both the LD50 and the maximum safe daily dosage for this substance. According to the findings of toxicological studies, glucomoringin does not have any mutagenic, carcinogenic, irritating, or reproductive effects, which indicates that it could be a viable option for safe medical treatment. The drug score of 0.3 indicates that the key pharmacological qualities, such as TPSA and drug-likeness, were found to be within acceptable levels. The evaluation of glucomoringin's bioactivity revealed that it possessed a high bioactive score in a number of significant therapeutic targets.

The treatment and management of Alzheimer's disease typically involve medications such as cholinesterase inhibitors Donepezil [^50-52^] and Rivastigmine [^53-56^]; NMDA receptor antagonist Memantine [^57-61^] to manage symptoms, support from caregivers and lifestyle interventions, but there is no cure for the disease. Promising areas of research include understanding the role of inflammation and oxidative stress in the development and progression of Alzheimer's disease, but more research is needed to develop effective treatments. While the compounds found in plants like moringa, including glucomoringin [62,63], may have various health benefits and antioxidant properties, they should not be considered as a sole or primary treatment for Alzheimer's disease. If you or someone you know is concerned about Alzheimer's disease, it is important to consult a healthcare professional for an accurate diagnosis and appropriate treatment options. Additionally, any potential use of natural compounds in treating or preventing Alzheimer's disease would require extensive clinical research and validation. glucomoringin is believed to have anti-inflammatory properties [^64-66^] due to its potential to inhibit the production of inflammatory molecules in the body. Inflammation is a natural response to injury or infection, but chronic inflammation is associated with various health problems, including chronic diseases. Some studies suggest that moringa extracts, which contain glucomoringin, may help reduce inflammation in animal models [67]. It is also considered an antioxidant. Antioxidants are compounds that help neutralize harmful free radicals in the body, which can cause oxidative stress and damage cells. By scavenging these free radicals, antioxidants can protect cells and tissues from damage. Moringa, with its various antioxidants, including glucomoringin, is thought to help combat oxidative stress [64,66]. It is important to understand that while there is potential for health benefits associated with glucomoringin; more research is needed to establish its exact mechanisms of action and its efficacy in humans. Furthermore, the concentration of glucomoringin in moringa products can vary, so its effects may depend on the source and preparation. Despite the fact that the findings are encouraging, additional empirical research needs to be conducted in order to evaluate its efficiency and safety for certain applications. However, additional clinical tests are required to evaluate whether or not they are safe and whether or not they are successful in treating Alzheimer's disease. When contemplating alternative treatments, it is essential to work together with medical specialists in order to develop a care plan that is both comprehensive and risk-free. Because it has such a wide range of bioactivity, it is an attractive option for further research into medication development.

## Future perspective

Advancements and challenges characterize research and treatment for Alzheimer's disease. Detecting the disease early is crucial, prompting current research to focus on accurate and accessible biomarkers for early-stage identification. Technologies such as neuroimaging, blood tests and wearable devices may aid in early diagnosis. The rise of personalized or precision medicine involves tailoring treatments to an individual's genetic and molecular profile, acknowledging Alzheimer's complexity and diverse causes. While current treatments manage symptoms, ongoing research aims to develop disease-modifying therapies targeting underlying causes like amyloid plaques and tau tangles to slow or halt progression. Combining various treatments, including drugs, lifestyle changes and alternative therapies, is under exploration for improved outcomes. Non-pharmacological interventions like cognitive training, exercise and diet gain attention for supporting cognitive health and delaying symptoms. Technology, such as digital health tools and telemedicine, is increasingly relevant in Alzheimer's care, offering remote monitoring, cognitive training apps and telehealth consultations. Collaboration among researchers, institutions and nations is vital for accelerating progress. Sharing data and findings helps identify trends and potential breakthroughs. The well-being of caregivers is a significant concern, emphasizing the need for support, education and resources. Future efforts should prioritize developing healthcare policies and systems to address the growing Alzheimer's burden, ensuring early diagnosis, affordable care and support for individuals and their families. Increasing public awareness and reducing Alzheimer's stigma are crucial for early diagnosis, research funding and supporting those affected by the condition.

## Limitation

Preliminary research findings form the basis for herbal treatments such as moringa and its alkaloids in addressing Alzheimer's disease. However, the efficacy and safety of these treatments remain uncertain, necessitating further clinical trials and research. Given the intricate nature of Alzheimer's disease with multiple contributing factors, it is crucial to acknowledge that there is no definitive cure. Treatment typically involves a combination of approaches, including medication, lifestyle adjustments and supportive care. It is imperative to discuss the use of herbal treatments with a healthcare professional due to variations in safety and efficacy based on individual health conditions, medications and other factors. Although herbal treatments show promise, they present challenges like inconsistent product quality, lack of standardized dosages and potential interactions with conventional medications. Rigorous clinical trials are indispensable for any treatment, including herbal remedies, to establish their safety and efficacy. Preliminary research findings should not be misconstrued as conclusive treatment options. Emphasizing the significance of consulting with a healthcare professional when considering Alzheimer's treatment is crucial. Self-medicating or relying solely on alternative therapies without medical guidance can pose risks. Alzheimer's disease lacks a one-size-fits-all treatment, underscoring the need for individualized care plans. Patients and their families should collaborate closely with healthcare providers to determine the most appropriate treatments and interventions.

## Conclusion

The *in silico* evaluation of glucomoringin as a potential therapeutic agent for Alzheimer's disease presents promising findings. Molecular docking assessments indicate its potential as an anti-inflammatory agent, particularly in targeting interleukin-1β. The compound's pharmacokinetic properties, including favorable CNS permeability, warrant further investigation into appropriate dosing strategies. Importantly, glucomoringin demonstrates a favorable safety profile, making it a compelling candidate for further empirical research and potential therapeutic applications in Alzheimer's disease. While these *in silico* findings provide valuable insights, empirical studies are necessary to validate its efficacy and safety for clinical use. glucomoringin stands as a promising natural compound in the pursuit of novel treatments for Alzheimer's disease. As research in Alzheimer's disease continues to advance, these findings contribute to the growing body of knowledge aimed at developing effective treatments for this devastating condition.
